# {*N*′-[(*E*)-1-(5-Bromo-2-oxidophen­yl)ethyl­idene]-4-chloro­benzohydrazidato}pyridinenickel(II)

**DOI:** 10.1107/S160053680902234X

**Published:** 2009-06-20

**Authors:** Xiu-Li Chang, Bin Xie, Chang-You Ji, Yang-Guang Xiang, Li-Ke Zou

**Affiliations:** a School of Chemistry & Pharmaceutical Engineering, Sichuan University of Science & Engineering, Zigong, Sichuan 643000, People’s Republic of China; b College of Environment and Chemical Engineering, Xi’an Polytechnic University, 710048 Xi’an, Shaanxi, People’s Republic of China

## Abstract

The title complex, [Ni(C_15_H_10_BrClN_2_O_2_)(C_5_H_5_N)], displays a square-planar coordination geometry around the Ni^II^ ion, formed by the tridentate hydrazone and monodentate pyridine ligands, with the N atoms in a *trans* arrangement about the Ni center.

## Related literature

For the coordination properties of aroylhydrazones, see: Ali *et al.* (2004 ▶); Carcelli *et al.* (1995 ▶); Salem (1998 ▶); Singh *et al.* (1982 ▶).
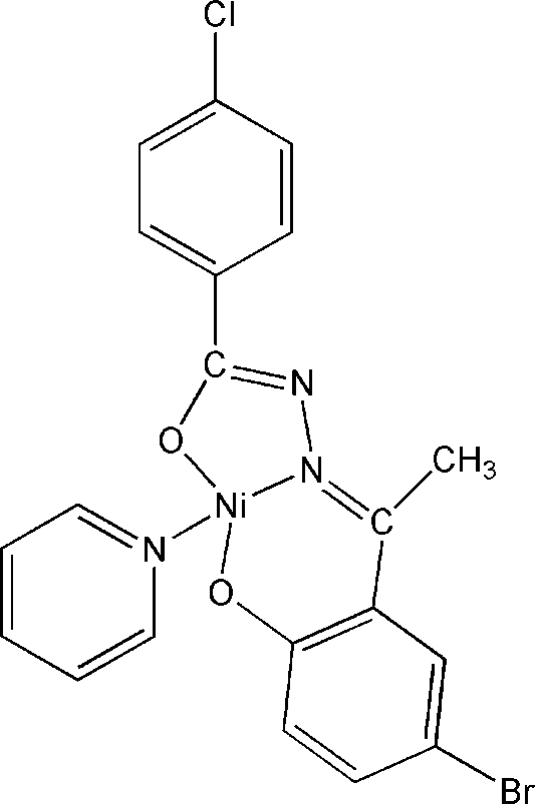


## Experimental

### 

#### Crystal data

[Ni(C_15_H_10_BrClN_2_O_2_)(C_5_H_5_N)]
*M* *_r_* = 503.42
Monoclinic, 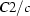

*a* = 32.430 (4) Å
*b* = 6.0816 (8) Å
*c* = 22.865 (3) Å
β = 121.422 (2)°
*V* = 3848.3 (9) Å^3^
*Z* = 8
Mo *K*α radiation
μ = 3.25 mm^−1^
*T* = 278 K
0.18 × 0.13 × 0.10 mm

#### Data collection

Siemens SMART CCD area-detector diffractometer
Absorption correction: multi-scan (*SADABS*; Sheldrick, 1996 ▶) *T*_min_ = 0.581, *T*_max_ = 0.723
9680 measured reflections
3401 independent reflections
2651 reflections with *I* > 2σ(*I*)
*R*_int_ = 0.028

#### Refinement

*R*[*F* ^2^ > 2σ(*F* ^2^)] = 0.032
*wR*(*F* ^2^) = 0.080
*S* = 1.02
3401 reflections
253 parameters
H-atom parameters constrained
Δρ_max_ = 0.48 e Å^−3^
Δρ_min_ = −0.27 e Å^−3^

### 

Data collection: *SMART* (Siemens, 1996 ▶); cell refinement: *SAINT* (Siemens, 1996 ▶); data reduction: *SAINT*; program(s) used to solve structure: *SHELXS97* (Sheldrick, 2008 ▶); program(s) used to refine structure: *SHELXL97* (Sheldrick, 2008 ▶); molecular graphics: *SHELXTL* (Sheldrick, 2008 ▶); software used to prepare material for publication: *SHELXTL*.

## Supplementary Material

Crystal structure: contains datablocks I, global. DOI: 10.1107/S160053680902234X/bh2230sup1.cif

Structure factors: contains datablocks I. DOI: 10.1107/S160053680902234X/bh2230Isup2.hkl

Additional supplementary materials:  crystallographic information; 3D view; checkCIF report

## Figures and Tables

**Fig. 1.**
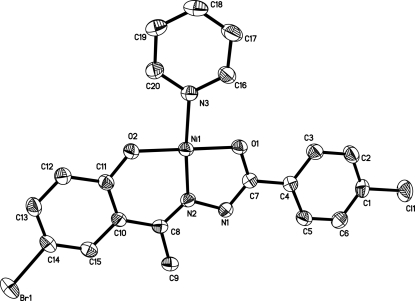
The molecular structure of the title compound, showing the atom-numbering scheme. Displacement ellipsoids are drawn at the 30% probability level. H atoms have been omitted.

## supplementary crystallographic information

### Comment

The chemistry of aroylhydrazones continues to attract much attention due to their coordination ability to metal ions (Singh *et al.*, 1982; Salem, 1998; Ali *et al.*, 2004) and their biological activity (Singh *et al.*, 1982; Carcelli *et al.*, 1995). As an extension of work on the structural characterization of aroylhydrazone derivatives, the title compound was synthesized and its crystal structure is reported.

### Experimental

A DMF solution (5 ml) of *N'*-[(*E*)-(5-bromo-2-hydroxyphenyl)ethylidene]-4-chlorobenzo-hydrazide (0.25 mmol, 0.092 g) was mixed with a methanol solution (5 ml) of NiCl_2_.6H_2_O (0.25 mmol, 0.059 g). The mixture was stirred at 298 K for 4 h and then filtered. A green precipitate was produced after about 10 d. A pyridine mixture (5 ml) was used to dissolve the precipitate at 330 K. Dark green block-shaped crystals were obtained after one month (yield 30%).

### Refinement

H atoms were placed in calculated positions, with C—H bond lengths fixed to 0.93 (aromatic CH) or 0.96 Å (methyl CH_3_). Isotropic displacement parameters for H atoms were computed as *U*_iso_(H) =*xU*_eq_(carrier C) with *x* = 1.2 (aromatic CH) or 1.5 (methyl CH_3_).

### Crystal data

**Table:**

[Ni(C_15_H_10_BrClNO_2_)(C_5_H_5_N)]	*F*(000) = 2016
*M**_r_* = 503.42	*D*_x_ = 1.738 Mg m^−^^3^
Monoclinic, *C*2/*c*	Melting point: 330 K
Hall symbol: -C 2yc	Mo *K*α radiation, λ = 0.71073 Å
*a* = 32.430 (4) Å	Cell parameters from 2958 reflections
*b* = 6.0816 (8) Å	θ = 2.6–25.0°
*c* = 22.865 (3) Å	µ = 3.25 mm^−^^1^
β = 121.422 (2)°	*T* = 278 K
*V* = 3848.3 (9) Å^3^	Block, green
*Z* = 8	0.18 × 0.13 × 0.10 mm

### Data collection

**Table:**

Siemens SMART CCD area-detector diffractometer	3401 independent reflections
Radiation source: fine-focus sealed tube	2651 reflections with *I* > 2σ(*I*)
graphite	*R*_int_ = 0.028
φ and ω scans	θ_max_ = 25.1°, θ_min_ = 1.5°
Absorption correction: multi-scan (*SADABS*; Sheldrick, 1996)	*h* = −37→38
*T*_min_ = 0.581, *T*_max_ = 0.723	*k* = −7→7
9680 measured reflections	*l* = −24→27

### Refinement

**Table:**

Refinement on *F*^2^	Primary atom site location: structure-invariant direct methods
Least-squares matrix: full	Secondary atom site location: difference Fourier map
*R*[*F*^2^ > 2σ(*F*^2^)] = 0.032	Hydrogen site location: inferred from neighbouring sites
*wR*(*F*^2^) = 0.080	H-atom parameters constrained
*S* = 1.02	*w* = 1/[σ^2^(*F*_o_^2^) + (0.0383*P*)^2^ + 2.3403*P*] where *P* = (*F*_o_^2^ + 2*F*_c_^2^)/3
3401 reflections	(Δ/σ)_max_ < 0.001
253 parameters	Δρ_max_ = 0.48 e Å^−^^3^
0 restraints	Δρ_min_ = −0.27 e Å^−^^3^
0 constraints	

### Fractional atomic coordinates and isotropic or equivalent isotropic displacement parameters (Å^2^)

**Table:**

	*x*	*y*	*z*	*U*_iso_*/*U*_eq_	
Ni1	0.041268 (13)	0.23786 (6)	0.156018 (18)	0.04192 (13)	
Br1	−0.211124 (13)	−0.09987 (7)	−0.01369 (2)	0.07519 (15)	
Cl1	0.26998 (4)	−0.39055 (19)	0.14682 (6)	0.0893 (3)	
O1	0.09933 (7)	0.1537 (3)	0.16792 (10)	0.0483 (5)	
O2	−0.01644 (7)	0.3235 (3)	0.14192 (11)	0.0532 (5)	
N1	0.05323 (9)	−0.1250 (4)	0.09609 (12)	0.0460 (6)	
N2	0.01755 (9)	−0.0048 (4)	0.09955 (11)	0.0418 (6)	
N3	0.07253 (9)	0.4809 (4)	0.22016 (12)	0.0449 (6)	
C1	0.21951 (12)	−0.2894 (6)	0.14580 (16)	0.0572 (9)	
C2	0.22207 (12)	−0.0906 (6)	0.17554 (17)	0.0647 (9)	
H2	0.2510	−0.0129	0.1980	0.078*	
C3	0.18122 (12)	−0.0071 (6)	0.17171 (16)	0.0578 (8)	
H3	0.1827	0.1285	0.1915	0.069*	
C4	0.13809 (11)	−0.1220 (5)	0.13888 (14)	0.0454 (7)	
C5	0.13736 (13)	−0.3249 (5)	0.11103 (18)	0.0595 (9)	
H5	0.1089	−0.4065	0.0899	0.071*	
C6	0.17748 (13)	−0.4080 (6)	0.11382 (18)	0.0646 (9)	
H6	0.1763	−0.5437	0.0942	0.077*	
C7	0.09448 (11)	−0.0276 (5)	0.13425 (14)	0.0437 (7)	
C8	−0.02649 (11)	−0.0808 (5)	0.06426 (13)	0.0417 (7)	
C9	−0.03642 (12)	−0.2861 (5)	0.02193 (16)	0.0539 (8)	
H9A	−0.0094	−0.3164	0.0169	0.081*	
H9B	−0.0649	−0.2649	−0.0225	0.081*	
H9C	−0.0413	−0.4077	0.0444	0.081*	
C10	−0.06526 (10)	0.0294 (5)	0.06629 (13)	0.0415 (7)	
C11	−0.05843 (11)	0.2266 (5)	0.10261 (15)	0.0456 (7)	
C12	−0.09895 (12)	0.3312 (5)	0.09768 (16)	0.0545 (8)	
H12	−0.0947	0.4649	0.1198	0.065*	
C13	−0.14427 (12)	0.2416 (6)	0.06125 (17)	0.0564 (8)	
H13	−0.1706	0.3138	0.0582	0.068*	
C14	−0.15003 (11)	0.0428 (6)	0.02932 (15)	0.0516 (8)	
C15	−0.11213 (11)	−0.0603 (5)	0.03060 (14)	0.0487 (7)	
H15	−0.1174	−0.1926	0.0074	0.058*	
C16	0.12054 (12)	0.4904 (6)	0.25973 (16)	0.0600 (9)	
H16	0.1387	0.3768	0.2571	0.072*	
C17	0.14431 (14)	0.6615 (6)	0.30425 (19)	0.0701 (10)	
H17	0.1779	0.6628	0.3311	0.084*	
C18	0.11809 (15)	0.8293 (6)	0.30861 (17)	0.0655 (10)	
H18	0.1335	0.9482	0.3375	0.079*	
C19	0.06898 (14)	0.8192 (5)	0.26974 (17)	0.0589 (9)	
H19	0.0503	0.9294	0.2727	0.071*	
C20	0.04720 (12)	0.6437 (5)	0.22580 (15)	0.0525 (8)	
H20	0.0136	0.6388	0.1992	0.063*	

### Atomic displacement parameters (Å^2^)

**Table:**

	*U*^11^	*U*^22^	*U*^33^	*U*^12^	*U*^13^	*U*^23^
Ni1	0.0420 (2)	0.0397 (2)	0.0444 (2)	−0.00392 (17)	0.02273 (18)	−0.00596 (17)
Br1	0.0486 (2)	0.0968 (3)	0.0797 (3)	−0.0210 (2)	0.0332 (2)	−0.0084 (2)
Cl1	0.0596 (6)	0.1029 (8)	0.1085 (8)	0.0179 (6)	0.0461 (6)	−0.0134 (6)
O1	0.0456 (12)	0.0462 (12)	0.0522 (12)	−0.0061 (9)	0.0248 (10)	−0.0130 (10)
O2	0.0433 (13)	0.0501 (12)	0.0657 (13)	−0.0055 (10)	0.0281 (11)	−0.0161 (11)
N1	0.0454 (15)	0.0431 (15)	0.0514 (15)	0.0007 (12)	0.0265 (13)	−0.0048 (12)
N2	0.0445 (15)	0.0388 (13)	0.0440 (13)	−0.0011 (11)	0.0245 (12)	−0.0025 (11)
N3	0.0486 (16)	0.0436 (14)	0.0464 (14)	−0.0058 (12)	0.0275 (12)	−0.0051 (11)
C1	0.047 (2)	0.067 (2)	0.058 (2)	0.0122 (17)	0.0272 (17)	0.0007 (17)
C2	0.044 (2)	0.074 (3)	0.074 (2)	−0.0059 (17)	0.0297 (18)	−0.0151 (19)
C3	0.054 (2)	0.057 (2)	0.067 (2)	−0.0062 (17)	0.0347 (18)	−0.0163 (17)
C4	0.0454 (18)	0.0493 (19)	0.0432 (16)	0.0000 (14)	0.0242 (15)	−0.0001 (14)
C5	0.050 (2)	0.054 (2)	0.072 (2)	−0.0075 (16)	0.0299 (18)	−0.0163 (17)
C6	0.065 (2)	0.056 (2)	0.078 (2)	0.0055 (18)	0.040 (2)	−0.0129 (18)
C7	0.0457 (18)	0.0448 (17)	0.0423 (16)	0.0001 (14)	0.0241 (15)	0.0003 (14)
C8	0.0465 (18)	0.0381 (16)	0.0388 (15)	−0.0049 (13)	0.0210 (14)	0.0000 (13)
C9	0.055 (2)	0.0444 (19)	0.0595 (19)	−0.0082 (15)	0.0274 (17)	−0.0105 (15)
C10	0.0414 (17)	0.0421 (17)	0.0425 (15)	−0.0046 (13)	0.0229 (14)	0.0012 (13)
C11	0.0441 (18)	0.0476 (18)	0.0494 (17)	−0.0047 (14)	0.0274 (15)	0.0001 (14)
C12	0.053 (2)	0.0525 (19)	0.064 (2)	−0.0029 (16)	0.0350 (17)	−0.0048 (16)
C13	0.047 (2)	0.066 (2)	0.066 (2)	0.0010 (17)	0.0358 (17)	0.0043 (18)
C14	0.0421 (18)	0.063 (2)	0.0496 (18)	−0.0102 (16)	0.0239 (15)	0.0023 (16)
C15	0.0504 (19)	0.0510 (18)	0.0465 (17)	−0.0095 (15)	0.0266 (15)	−0.0020 (14)
C16	0.048 (2)	0.064 (2)	0.070 (2)	−0.0108 (17)	0.0320 (18)	−0.0193 (18)
C17	0.059 (2)	0.075 (2)	0.074 (2)	−0.025 (2)	0.033 (2)	−0.028 (2)
C18	0.085 (3)	0.059 (2)	0.062 (2)	−0.028 (2)	0.045 (2)	−0.0210 (18)
C19	0.079 (3)	0.0463 (19)	0.059 (2)	−0.0047 (18)	0.042 (2)	−0.0088 (16)
C20	0.056 (2)	0.0511 (19)	0.0510 (18)	−0.0009 (16)	0.0288 (17)	−0.0042 (15)

### Geometric parameters (Å, °)

**Table:**

Ni1—O2	1.802 (2)	C8—C10	1.447 (4)
Ni1—O1	1.830 (2)	C8—C9	1.508 (4)
Ni1—N2	1.844 (2)	C9—H9A	0.9600
Ni1—N3	1.953 (2)	C9—H9B	0.9600
Br1—C14	1.901 (3)	C9—H9C	0.9600
Cl1—C1	1.737 (3)	C10—C15	1.407 (4)
O1—C7	1.306 (3)	C10—C11	1.409 (4)
O2—C11	1.317 (3)	C11—C12	1.410 (4)
N1—C7	1.298 (4)	C12—C13	1.369 (4)
N1—N2	1.405 (3)	C12—H12	0.9300
N2—C8	1.305 (4)	C13—C14	1.374 (4)
N3—C16	1.333 (4)	C13—H13	0.9300
N3—C20	1.335 (4)	C14—C15	1.366 (4)
C1—C2	1.368 (5)	C15—H15	0.9300
C1—C6	1.369 (5)	C16—C17	1.377 (4)
C2—C3	1.378 (4)	C16—H16	0.9300
C2—H2	0.9300	C17—C18	1.364 (5)
C3—C4	1.383 (4)	C17—H17	0.9300
C3—H3	0.9300	C18—C19	1.362 (5)
C4—C5	1.383 (4)	C18—H18	0.9300
C4—C7	1.478 (4)	C19—C20	1.381 (4)
C5—C6	1.366 (5)	C19—H19	0.9300
C5—H5	0.9300	C20—H20	0.9300
C6—H6	0.9300		
			
O2—Ni1—O1	178.39 (9)	C8—C9—H9A	109.5
O2—Ni1—N2	95.20 (10)	C8—C9—H9B	109.5
O1—Ni1—N2	84.35 (9)	H9A—C9—H9B	109.5
O2—Ni1—N3	89.99 (10)	C8—C9—H9C	109.5
O1—Ni1—N3	90.52 (9)	H9A—C9—H9C	109.5
N2—Ni1—N3	174.43 (11)	H9B—C9—H9C	109.5
C7—O1—Ni1	110.50 (18)	C15—C10—C11	117.5 (3)
C11—O2—Ni1	126.92 (19)	C15—C10—C8	119.7 (3)
C7—N1—N2	108.4 (2)	C11—C10—C8	122.7 (3)
C8—N2—N1	116.7 (2)	O2—C11—C10	124.9 (3)
C8—N2—Ni1	129.7 (2)	O2—C11—C12	116.2 (3)
N1—N2—Ni1	113.61 (18)	C10—C11—C12	118.9 (3)
C16—N3—C20	117.5 (3)	C13—C12—C11	122.0 (3)
C16—N3—Ni1	120.5 (2)	C13—C12—H12	119.0
C20—N3—Ni1	122.0 (2)	C11—C12—H12	119.0
C2—C1—C6	121.1 (3)	C12—C13—C14	118.6 (3)
C2—C1—Cl1	119.6 (3)	C12—C13—H13	120.7
C6—C1—Cl1	119.3 (3)	C14—C13—H13	120.7
C1—C2—C3	119.1 (3)	C15—C14—C13	121.4 (3)
C1—C2—H2	120.4	C15—C14—Br1	119.0 (3)
C3—C2—H2	120.4	C13—C14—Br1	119.5 (2)
C2—C3—C4	121.0 (3)	C14—C15—C10	121.5 (3)
C2—C3—H3	119.5	C14—C15—H15	119.3
C4—C3—H3	119.5	C10—C15—H15	119.3
C3—C4—C5	118.1 (3)	N3—C16—C17	122.7 (3)
C3—C4—C7	120.1 (3)	N3—C16—H16	118.6
C5—C4—C7	121.8 (3)	C17—C16—H16	118.6
C6—C5—C4	121.4 (3)	C18—C17—C16	119.3 (4)
C6—C5—H5	119.3	C18—C17—H17	120.4
C4—C5—H5	119.3	C16—C17—H17	120.4
C5—C6—C1	119.2 (3)	C19—C18—C17	118.7 (3)
C5—C6—H6	120.4	C19—C18—H18	120.7
C1—C6—H6	120.4	C17—C18—H18	120.7
N1—C7—O1	123.1 (3)	C18—C19—C20	119.4 (3)
N1—C7—C4	118.9 (3)	C18—C19—H19	120.3
O1—C7—C4	118.0 (3)	C20—C19—H19	120.3
N2—C8—C10	120.4 (3)	N3—C20—C19	122.4 (3)
N2—C8—C9	119.3 (3)	N3—C20—H20	118.8
C10—C8—C9	120.4 (3)	C19—C20—H20	118.8
			
N2—Ni1—O1—C7	−2.06 (18)	N1—N2—C8—C10	178.4 (2)
N3—Ni1—O1—C7	175.76 (18)	Ni1—N2—C8—C10	−0.2 (4)
N2—Ni1—O2—C11	0.0 (2)	N1—N2—C8—C9	−1.0 (4)
N3—Ni1—O2—C11	−178.0 (2)	Ni1—N2—C8—C9	−179.5 (2)
C7—N1—N2—C8	−179.6 (2)	N2—C8—C10—C15	−177.0 (2)
C7—N1—N2—Ni1	−0.8 (3)	C9—C8—C10—C15	2.4 (4)
O2—Ni1—N2—C8	−1.3 (3)	N2—C8—C10—C11	3.4 (4)
O1—Ni1—N2—C8	−179.8 (3)	C9—C8—C10—C11	−177.3 (3)
O2—Ni1—N2—N1	−179.92 (18)	Ni1—O2—C11—C10	2.9 (4)
O1—Ni1—N2—N1	1.62 (17)	Ni1—O2—C11—C12	−177.3 (2)
O2—Ni1—N3—C16	168.9 (2)	C15—C10—C11—O2	175.5 (3)
O1—Ni1—N3—C16	−12.6 (2)	C8—C10—C11—O2	−4.9 (4)
O2—Ni1—N3—C20	−11.7 (2)	C15—C10—C11—C12	−4.3 (4)
O1—Ni1—N3—C20	166.7 (2)	C8—C10—C11—C12	175.4 (3)
C6—C1—C2—C3	−1.5 (5)	O2—C11—C12—C13	−176.7 (3)
Cl1—C1—C2—C3	177.0 (3)	C10—C11—C12—C13	3.1 (5)
C1—C2—C3—C4	0.5 (5)	C11—C12—C13—C14	0.7 (5)
C2—C3—C4—C5	1.1 (5)	C12—C13—C14—C15	−3.2 (5)
C2—C3—C4—C7	−178.9 (3)	C12—C13—C14—Br1	173.6 (2)
C3—C4—C5—C6	−1.9 (5)	C13—C14—C15—C10	1.9 (5)
C7—C4—C5—C6	178.1 (3)	Br1—C14—C15—C10	−175.0 (2)
C4—C5—C6—C1	1.0 (5)	C11—C10—C15—C14	2.0 (4)
C2—C1—C6—C5	0.7 (5)	C8—C10—C15—C14	−177.7 (3)
Cl1—C1—C6—C5	−177.8 (3)	C20—N3—C16—C17	−1.3 (5)
N2—N1—C7—O1	−1.1 (4)	Ni1—N3—C16—C17	178.0 (3)
N2—N1—C7—C4	−179.6 (2)	N3—C16—C17—C18	0.0 (5)
Ni1—O1—C7—N1	2.4 (3)	C16—C17—C18—C19	1.5 (5)
Ni1—O1—C7—C4	−179.08 (19)	C17—C18—C19—C20	−1.8 (5)
C3—C4—C7—N1	171.6 (3)	C16—N3—C20—C19	1.0 (4)
C5—C4—C7—N1	−8.4 (4)	Ni1—N3—C20—C19	−178.3 (2)
C3—C4—C7—O1	−7.0 (4)	C18—C19—C20—N3	0.5 (5)
C5—C4—C7—O1	173.0 (3)		
